# Complications in groin hernia surgery and the way out

**DOI:** 10.4103/0972-9941.27734

**Published:** 2006-09

**Authors:** Pradeep K Chowbey, Murtaza Pithawala, Rajesh Khullar, Anil Sharma, Vandana Soni, Manish Baijal

**Affiliations:** Minimal Access and Bariatric Surgery Centre, Sir Ganga Ram Hospital, New Delhi - 110 060, India

**Keywords:** Endoscopic, Inguinal, hernia, complications

## Abstract

Complications in endoscopic inguinal hernia surgery are more dangerous and more frequent than those of open surgery, especially in inexperienced hands and hence are best avoided. It is possible to avoid most of these complications if one follows a set of well-defined steps and principles of endoscopic inguinal hernia surgery.

Complications are known to occur at each and every step of hernia surgery. Applying caution while performing each step can save the patient from a lot of morbidity. One starts by applying strict patient selection criteria for endoscopic hernia repair, especially in the initial part of ones learning curve. A thorough knowledge of anatomy goes a long way in avoiding most of the complications seen in hernia repair. This anatomy needs to be relearned from what one is used to, as the approach is totally different from an open hernia repair. And finally, learning and mastering the right technique is an essential prerequisite before one ventures into inguinal hernia repair.

Although there has been an increased incidence of complications reported in endoscopic repair in the earlier series, this can be explained partly by the fact that it was in the early part of the learning curve of most endoscopic surgeons. As the experience grew and the techniques were standardized, the incidences of complications have also reduced and have come to be on par with open hernia surgery. The various complications and precautions to be taken to avoid them will be discussed.

In inexperienced hands, complications in laparoscopic inguinal hernia surgery are more dangerous and more frequent than those of open surgery and hence are best avoided. It is possible to avoid most of these complications if one follows a set of well-defined steps and principles of laparoscopic surgery. The incidence of complications has fallen as the experience has grown and it has proven itself to be a safe procedure in the hands of experienced surgeons.

Here we shall discuss various complications, as well as the steps and precautions to be taken to avoid them.

Complications and the various precautions to be taken in hernia surgery can be divided into:

1. Preoperative
2. Intraoperative
3. Postoperative

## PREOPERATIVE PRECAUTIONS

The most important preoperative precaution is proper patient selection prior to surgery, especially in the initial part of the learning curve. Ideally, direct or small indirect hernias are best. Large hernias, obese patients and irreducible, obstructed hernias are best avoided. An absolute contraindication is strangulated hernia. Also a detailed work-up of elderly patients to assess cardiorespiratory status is mandatory to ensure a safe outcome.

## INTRAOPERATIVE COMPLICATIONS AND PRECAUTIONS

### 1) During creation of preperitoneal space

This is the most important step for beginners.

- A wide linea alba may result in breaching the peritoneum; in such a situation, it is best to close the rectus and incise the sheath more laterally
- Improper placement of balloon trocar causing dissection of muscle fibers
- Entry into peritoneum causing pneumoperitoneum
- Rupture of balloon in preperitoneal space
- The Hassan's trocar must snugly fit into the incision to avoid CO_2_ leak

To avoid these, one must ensure that the balloon is made properly and the correct space is entered by retracting the rectus muscle laterally to visualize the posterior rectus sheath. Also the balloon trocar is inserted gently, parallel to the abdominal wall, to avoid puncturing the peritoneum. The balloon must be inflated slowly with saline to ensure smooth and even distension and prevent its rupture.

### 2) Precautions during port placement

The trocars should be short and threaded in proportion to less workspace and to ensure a snug fit respectively. The skin incisions should be just adequate to grip the trocar and prevent its slipping. The patient should empty their bladder before surgery as the suprapubic trocar could injure a filled bladder. The pressure in the preperitoneal space must be such as to offer sufficient resistance during trocar insertion to avoid puncturing the peritoneum.

### 3) Correct identification of the anatomical landmarks

The next most important and crucial step in any hernia surgery is the correct identification of anatomical landmarks. This is difficult for beginners as the anatomy is different from that seen in open surgery. The first most important step is to identify the pubic bone. Once this is seen, the rest of the landmarks are traced keeping this as reference point. One is advised to keep away from the triangle of doom, which contains the iliac vessels and to avoid placing tacks in the triangle of pain laterally.

### 4) Bladder injury

Bladder injury most commonly occurs during port placement, dissecting a large direct sac or in a sliding hernia. It is mandatory to empty the bladder prior to an inguinal hernia repair to avoid a trocar injury. It is advisable that beginners catheterize the bladder during the initial part of their learning curve. The diagnosis is evident when one sees urine in the extraperitoneal space. Repair is done with vicryl in two layers and a urinary catheter inserted for 7–10 days.

### 5) Bowel injury

It is rare during hernia surgery. It can occur when reducing large hernias, inadvertent opening of peritoneum causing the bowel to come into the field of surgery and in reduction of sliding hernias. Injury is best avoided in such circumstances by opening the hernial sac as close as possible to the deep ring. The initial studies[1,2] showed a higher incidence, especially with TAPP, but it decreased over time.

### 6) Vascular injury

This is one of the commonest injuries occurring in hernia repair and often a reason for conversion. The various sites where it can occur is rectus muscle vessel injury during trocar insertion; inferior epigastric vessel injury; bleeding from venous plexus on the pubic symphysis; aberrant obturator vein injury; testicular vessel injury; and the most disastrous of all, iliac vessels, which requires an emergency conversion to control the bleeding and the immediate services of a vascular surgeon to repair the same. Most of the other bleeding can be controlled with cautery or clips. Careful dissection and adherence to the principles of surgery will help in avoiding most of these injuries.

### 7) Injury to vas deferens

Injury occurs while dissecting the hernial sac from the cord structures. The injury causes an eventual fibrotic narrowing of the vas. A complete transection of the vas needs to be repaired in a young patient. An injury to the vas is best avoided and this may be done by identifying before dividing any structure near the deep ring or floor of the extraperitoneal space. Also the separation of cord structures from the hernial sac must be gentle and direct; grasping of vas deferens with forceps must be avoided.

### 8) Pneumoperitoneum

It is a common occurrence in TEP which every surgeon should be prepared to handle. Putting the patient in Trendelenberg position and increasing the insufflation pressures to 15 mmHg helps. If the problem still persists, a Veress needle can be inserted at Palmer's point.

### Postoperative complications

#### 1) Seroma / hematoma formation

It is a common complication after laparoscopic hernia surgery, the incidence being in the range of 5–25%. They are specially seen after large indirect hernia repair. Most resolve spontaneously over 4–6 weeks. A seroma can be avoided by minimizing dissection of the hernial sac from the cord structures, fixing the direct sac to pubic bone and fenestrating the transversalis fascia in a direct hernia. Some surgeons put in a drain if there is excessive bleeding or after extensive dissection.

#### 2) Urinary retention

This complication after hernia repair has a reported incidence of 1.3 to 5.8%.[3,4] It is usually precipitated in elderly patients, especially if symptoms of prostatism are present. These patients are best catheterized prior to surgery and catheter removed the next day morning.

#### 3) Neuralgias

The incidence of this complication is reported to be between 0.5 and 4.6% depending on the technique of repair. The intraperitoneal onlay mesh method had the highest incidence of neuralgias in one study and was hence abandoned as a form of viable repair.[3] The commonly involved nerves are lateral cutaneous nerve of thigh, genitofemoral nerve and intermediate cutaneous nerve of thigh. They are usually involved by mesh-induced fibrosis or entrapment by a tack. The complication is prevented by avoiding fixing the mesh lateral to the deep inguinal ring in the region of the triangle of pain, safe dissection of a large hernial sac and no dissection of fascia over the psoas.

#### 4) Testicular pain and swelling

It occurs due to excessive dissection of a sac from the cord structures, especially a complete sac. Reported incidence is of 0.9 to 1.5%. Most are transient. Orchitis was found in a small number of patients[3,4] but did not lead to testicular atrophy.

#### 5) Mesh infection and wound infection

Wound infection rates are very low. Mesh infection is a very serious complication and care must be taken to maintain strict aseptic precautions during the entire procedure. Any endogenous infection must be treated with an adequate course of antibiotics prior to surgery.

#### 6) Recurrence

It is the most important endpoint of any hernia surgery. It requires a proper and thorough knowledge of anatomy and a thorough technique of repair to help keep the recurrence in endoscopic repair to a minimum.

The important points to be kept in mind during the surgery are:

- After dissecting direct sac, all peritoneal adhesions around the margin of the defect should be meticulously lysed.
- Always search for an indirect sac, even if a direct hernia has been reduced.
- Reflect the peritoneum off the cord completely
- Place an adequate size mesh to cover the myopectineal orifice completely, preferably the size of 15 × 15 cm.
- The lower margin of the mesh must be comfortably placed - medially in the retropubic space and laterally over the psoas muscle.
- Perform a 2-point fixation of the mesh on the medial aspect over the Cooper's ligament.
- Avoid cutting of the mesh over the cord. This weakens the mesh and provides a potential site for recurrence.
- Ensure adequate hemostasis prior to placing the mesh.
- The most important factor is the adequate training and learning of the right technique.

## CONCLUSIONS

Endoscopic hernia repair is a technique that is to soon become an important part of the armamentarium of a hernia surgeon. Although the initial reported incidence of complications following laparoscopic hernia repair was higher, it has fallen following the standardization of the technique. Felix *et al* found a complication rate of 6% over a 6-year period but when it was broken down into two 3-year periods, their rate was 3.6% in the first 3 years and dropped to 0.5% in the following 3 years.[5] Most long-term studies have shown a low incidence of morbidity following an endoscopic repair. By properly selecting a patient and by having a thorough knowledge of anatomy and adequate experience, one can safely perform endoscopic repair keeping the complications to a minimum.
